# Syndromes or Flexibility: Behavior during a Life History Transition of a Coral Reef Fish

**DOI:** 10.1371/journal.pone.0084262

**Published:** 2013-12-27

**Authors:** James R. White, Mark I. McCormick, Mark G. Meekan

**Affiliations:** 1 School of Marine and Tropical Biology, James Cook University, Townsville, Queensland, Australia; 2 Australian Institute of Marine Science, Botany Building, The University of Western Australia, Crawley, Western Australia, Australia; Tulane University Medical School, United States of America

## Abstract

The theory of behavioral syndromes focuses on quantifying variation in behavior within and among individual organisms and attempts to account for the maintenance of differences in behavior that occur in a consistent manner among individuals. Behavioral syndromes have potentially important ecological consequences (e.g. survivorship tradeoffs) and can be shaped by population dynamics through selective mortality. Here, we search for any evidence for consistency of behavior across situations in juveniles of a common damselfish, *Pomacentrus amboinensis* (Pomacentridae) at the transition between larval habitats in the plankton and juvenile habitats on the reef. Naïve fish leaving the pelagic phase to settle on reefs were caught by light traps and their behaviors observed using similar methods across three different situations (small aquaria, large aquaria, field setting); all of which represent low risk and well-sheltered environments. Seven behavioral traits were compared within and among individuals across situations to determine if consistent behavioral syndromes existed. No consistency was found in any single or combination of behavioral traits for individuals across all situations. We suggest that high behavioral flexibility is likely beneficial for newly-settled fish at this ontogenetic transition and it is possible that consistent behavioral syndromes are unlikely to emerge in juveniles until environmental experience is gained or certain combinations of behaviors are favored by selective mortality.

## Introduction

The study of individual consistency in animal behavior has attracted recent attention from researchers because of the potential for this variability to reflect underlying processes influencing an animal’s responses to a range of situations and for it to have significant effects on fitness [1], [2]. Because variation in behavioral traits is so widespread taxonomically, an understanding of flexibility in these traits has important implications for the ecology and evolution of different species [1], [3]–[6] and for predictions of how they will respond to environmental and ecological shifts [1], [7].

Sih and Bell (2008) define within-individual consistency as occurring when an individual behaves in a consistent way over time or across situations (e.g. absence vs. presence of predators) [8]. Between-individual consistency occurs when there is a correlation between behavioral traits (e.g. boldness and aggression) among individuals. Having behavioral consistency either within- and between-individuals is termed a behavioral syndrome [1], [8]. Individual consistency in one behavioral trait (e.g. activity rate in different situations) is referred to as a behavioral type [1].

Most previous research has focused on explaining why individual variation in behavioral traits exists and why there should be consistency within an individual over time. The former question deals with factors maintaining variation in behavior among individuals from the same population, while the latter focuses on factors that maintain stability in behavioral traits [2]. However, inconsistent use of terminology and methodology in previous work has led to similar behavioral traits being defined differently or distinct traits defined as equivalent across various animal behavioral studies, making comparisons difficult [9], [10]. Furthermore, there is a lack of fish studies that demonstrate consistent patterns of individual behavior by showing that multiple behavioral traits are correlated across multiple situations (especially in laboratory vs. natural settings). Additionally, few studies have used identical measures of behavior across situations. Perhaps unsurprisingly, these studies conclude that their target species showed little consistency in behavioral traits [11], [12]. Finally, a majority of fish behavioral studies have been conducted in the laboratory [10] on captive or captive-bred populations [13]. Although this is done to control for factors that could potentially confound results, it is extraordinarily difficult to create environments that approximate natural situations in the laboratory. Consequently, such studies assume that behavior of an animal in an artificial setting will be representative of its natural state. This assumption is rarely tested in the field [14].

Behavior can be influenced selectively by a wide variety of abiotic (temperature, illumination, habitat; [14]–[16] or biotic factors (hunger, thirst, stress; [14], [15]) that can vary significantly through ontogeny [17]. These factors, along with experience gained as an individual grows, can lead to situations where it is beneficial for an individual to change their behavior to adjust to conditions (behavioral flexibility) or risk elimination from the population (selective mortality) [14]. Group behavior also can influence changes in individual behavior. For example, bold rainbow trout (*Onchorhyncus mykiss*) reduced boldness after observing shy conspecifics [15]. The outcomes of research on associations between behavioral traits and behavioral flexibility [13] have been inconsistent, although some studies have suggested that a tendency to display bold behavior increases an individual’s ability to solve novel tasks [18], [19], while others have shown that individuals that are more shy and unaggressive have more behavioral flexibility [20].

Maintaining a certain degree of behavioral flexibility to suit changing environmental conditions is likely to be necessary for animals with complex life cycles that undergo life history transitions. The transition between these planktonic and benthic environments is a major developmental landmark for most coral reef fishes and makes these ideal organisms on which to investigate the relationship between adaptive behavioral traits and biotic factors. Furthermore, young reef fish arriving from the plankton into benthic habitats have no experience of their new environment. As such they make a useful model organism because these naïve juveniles enable researchers to control for learning behaviors through experience and examine behavioral consistency precisely at the time of settlement, which is a critical ontogenetic boundary and mortality bottleneck [21]–[25]. In this phase of their life cycle, reef fishes typically experience high mortality [26], with rates within the first 48 hours averaging 57% [26], [27] but can be >90% [23], [28]. Behavioral decisions at early settlement can thus influence survival and possibly the structure of reef communities [23], [25]. Thus, we expect juvenile reef fish to quickly adopt consistency in behaviors that are likely to influence survival at this life stage (e.g. boldness and aggression; [21]).

Our study examines whether naïve juveniles of the Ambon damselfish (*Pomacentrus amboinensis*) display consistent behavioral traits across three low-risk situations (i.e. whether they possessed behavioral syndromes). Because the same behavioral measures are used across different-sized laboratory arenas and within the field, this is one of the first studies to compare behaviors of fish observed in the laboratory with those in a natural setting. Furthermore, we investigated the role of an environmental factor (temperature) in influencing flexibility in behavioral traits. The relationships among behavioral traits across situations were also examined. If newly-settled fish display behavioral syndromes, then theory would predict that individuals should maintain a similar ranking of behavioral types (e.g. boldness) among situations.

## Methods

### Ethics Statement

This study was carried out in strict accordance with the recommendations under James Cook University (JCU) ethics protocols and approved by the JCU Animal Ethics Committee (Permit Number: A1067). All efforts were made to minimize animal handling and stress. Fish and coral collection was permitted by the Great Barrier Reef Marine Park Authority (Permit Number: G10/33784.1). Fish were collected using light traps and kept in flow through aquaria for the duration of the study where they were observed visually and subsequently returned to the field upon completion.

### Study Site and Species

Field experiments were conducted in the shallow coral habitats (2–4 m depths) at the back-reef of Lizard Island in the northern Great Barrier Reef (GBR) (14°40′S, 145°28′E). Juvenile *P.* *amboinensis* settle from the plankton at night to a variety of habitats in the northern GBR [29] with the greatest densities found on small reef patches at the base of shallow (<10 m depth) reefs. *P.* *amboinensis* has a pelagic larval duration of 15–23 days and settles from 10.3–15.1 mm standard length [30]. The juvenile body is mostly complete at settlement; however fish go through a rapid change in body pigmentation in less than 12 hours after settlement [31]. Previous studies have shown *P. amboinensis* is relatively site-attached [32] and moves only small distances (<1 m) during the first few months after settlement. Also, these damselfish can be collected immediately prior to the end of their larval phase before settling on the reef and thus are largely naïve to reef-based predators and behaviors learned after settlement. As experience can influence the behavioral phenotypes a fish will exhibit, using reef-naïve individuals reduces the variability that may result from markedly different experience histories. Due to its high abundance, small size, rapid development, and sedentary nature after settlement, *P. amboinensis* is an ideal model species for field observations and laboratory manipulations [24].

Similar studies with *P. amboinensis* and *P. wardi* found individuals displayed consistent behaviors over multiple sampling periods across short time scales after settlement in the field (1 to 3 d; White et al., *in prep.*, [23]). This study examines if the behavioral consistency remains when individuals are observed within different situations.

### Collection

We collected late-stage pelagic larvae of *P. amboinensis* using moored light traps (see small light trap in Figure 1 of [33]for design) during January 2010. Fish caught in similar traps have been used in numerous published behavioral studies and individuals characteristically display considerable among-individual variability in behavioral traits such as boldness [21]. Traps were anchored approximately 100 m from the nearest reef in ∼16 m of water at dusk and left overnight. Catches were emptied from the traps the next morning at 0730–0800 hours. Fish collected from the traps were transported to the laboratory where *P. amboinensis* was separated from all other species and maintained in a 30 L aquarium of aerated seawater. Individual fish were then assigned a label and placed in separate small aquaria (13 L) for 24 h to acclimatize to laboratory conditions before experiments began during which time they were fed *Artemia* nauplii twice during daylight hours 1.5 mL^−1^; ∼ 768 nauplii per fish per feeding). Fish were fasted for 12 h prior to initial observations. Seawater was unfiltered, therefore potentially contained food, although no feeding behaviors were observed outside of standard feeding times.

**Figure 1:**
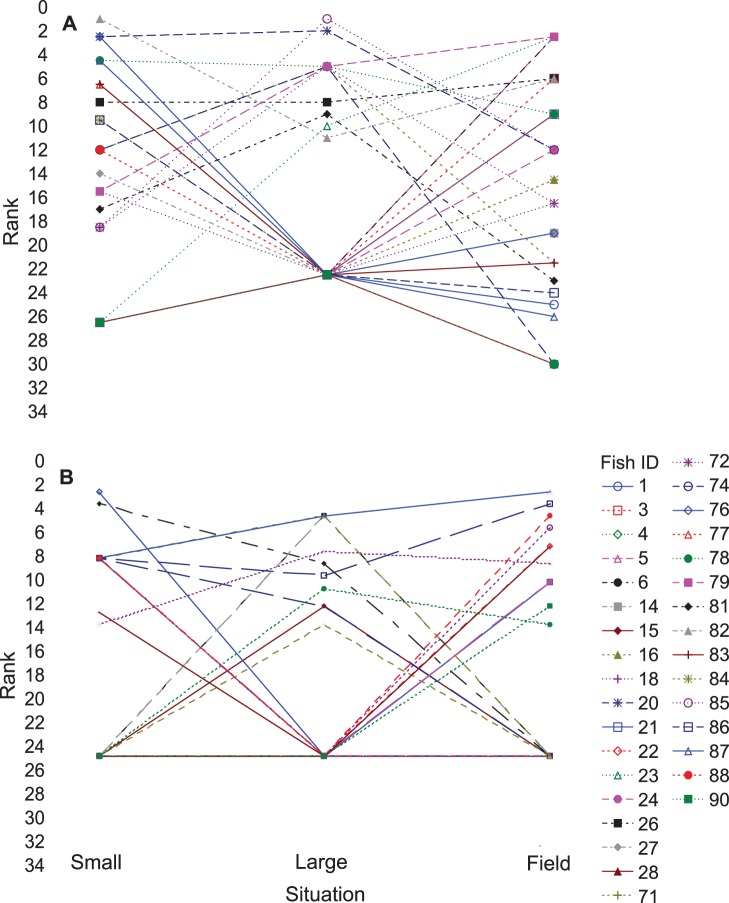
Rank order of boldness and height across situations. A) Ranking of boldness and B) ranking of height across small tanks, large tanks and field site). Each line and unique symbol represents individual fish (N = 33). Individuals were ranked sequentially according to the individual’s observed behavioral trait (1 = highest recorded value). Average ranking scores were assigned when multiple fish had a tie in values.

### Situations

Standard behavioral observations (below) were conducted on each individual fish under three different environmental situations: small aquaria (13 L, 20 cm water depth), large aquaria (285 L, 25 cm water depth) and shallow reef (field). All were established so they represented a simple low-risk, resource-rich environment with no competitors.

Firstly, behaviors of focal fish were observed in small aquaria (13 L) after 24 h acclimatization. Short pieces of PVC tubing (approx. 4 cm diameter) in each tank provided a refuge for each fish. Observers viewed fish through slits cut into a blind (black plastic sheet) to reduce any effect of observer presence. Barriers of black plastic sheeting prevented individuals from seeing fish in neighboring tanks.

After behavioral observations in the small tanks, individual fish were then transferred to the center of large circular tanks (285 L) and given at least a 20 min acclimation period before another series of behavioral observations. A short piece of PVC tubing (approx. 4 cm diameter) and replica low complexity artificial coral (white molded resin branching coral, item no. 21505; Wardleys/TFH, Sydney; dimensions 14×11.5×5 cm) provided refuge for each fish. Observations were made from a distance of at least 1.5 m from the tank and fish took no apparent notice of observers.

A final series of observations of the same fish was made in the field. Each *P. amboinensis* was placed into a labeled 2 L clip-seal plastic bag containing aerated seawater and transported to the field. One fish was released onto each reef of an array of small (30×30×30 cm) patch reefs of live bushy hard coral (*Pocillopora damicornis*) on a shallow sand flat. Reefs were positioned 5 m apart and ∼20 m from the nearest area of natural reef to avoid the re-dispersal of fish among reefs or colonization from natural reef. *P. amboinensis* naturally settles to this habitat. Prior to release of focal fish, patch reefs were cleared of all resident fishes using hand nets. Once fish were released, small wire cages (about 40×40×40 cm, 12 mm mesh size) were placed over the patch to allow the fish to acclimate to the new surroundings while being protected from predation. Cages were left a minimum of 20 min and removed (carefully lifted with slow movements) immediately before observations. Divers conducted observations with the aid of a 2× magnifying glass from at least 1 m away to avoid any observer affects that may have been caused by close proximity to the target fish. This protocol has been used in other behavioral studies and has not been found to disturb fish or alter their behavior in any significant way [21], [23], [24], [34].

While there were differences in habitat (e.g. variation in refuge size in different-sized arenas) and acclimation times among situations (small and large tanks and the field) these were mostly unavoidable trade-offs associated with logistical and efficiency issues typical of any empirical study. Once given time to de-stress after initial capture, the juvenile fish are generally quick to adapt to new situations, hence the shorter acclimation times for the large tank and field situations. Each situation resulted in different conditions for the subject, but all provided a living space, shelter, and an absence of competition and larger predators. Also, the same testing stimuli were presented to the focal fish in all settings. The way the fish responds to the stimulus will be a product of how it behaves in its environment (its perception of risk is likely to be different between situations) and this will influence how the fish responds to the stimulus. There are many things that are associated with the situations that are different that may affect fish behavior. But, this is the main aim of this study; given the difference in the environments, do fishes respond in consistent ways.

In addition to behavioral data, water temperature in the field and laboratory was recorded every 20 min using calibrated data loggers (32K StowAway Tidbit).

### Behavioral Observations

Identical measurements of behavior were made on each fish under the three settings (small & large aquaria, field). Fish were fasted for 12 hours before initial observations and were observed in all three situations on the same day in order to minimize the behavioral influence of individual metabolism or food availability. We recorded: bite rate (the number of strikes towards objects floating in the water column during 3 min); distance moved (the total distance covered in cm during 3 min); distance ventured as the percentage of time spent at various distances from refuge (e.g. 90% of time spent 2 cm away, 10% of time 5 cm away etc.); height from substrate (an estimate of percentage of time spent in either the upper, middle, or lower third of the coral patch); aggression recorded as mirror strike rate (after the initial 3 min observation a mirror was carefully placed in front of the fish and the combined number of strikes, tail whips, or aggressive displays made toward their reflection over 3 min was recorded [35], [36]; boldness (see below); latency (see below) to emerge from shelter after stimulus by a novel object (see below). The initial 7 behaviors were recorded during a 3 min observation, aggression was recorded in a separate 3 min trial, while boldness and latency were recorded in a separate 10 minute trial.

Boldness was defined as a continuous variable on a 0–3 scale, where 0 was hiding in a refuge before or immediately after introduction of a novel object and seldom emerging afterwards; 1 was retreating to refuge after a threat and taking more than 5 sec to re-emerge, then tentatively striking at food; 2 was retreating to refuge after a threat but emerging quickly and tenaciously striking at food; 3 was not hiding but continuing to explore or strike at food aggressively. This scale was similar to other measures of boldness used in earlier studies and has been shown to be consistent over short time periods (hours) within individuals, normally distributed, and related to survival [21], [23], [24], [34]. The boldness score basically establishes a spectrum of behavioral reaction to a stimulus and records where an individual lies within this spectrum.

Latency to emerge from hiding was recorded as the amount of time it took the fish to leave their refuge after introduction of a novel object. This variable was limited to a 10 min observation time. In both boldness and latency measures, the novel object was a lead bean sinker weight that was tied to clear fishing line and dropped from 1 m above the refuge in each setting. In the small and large aquariums, short sections of PVC pipe were suspended above each tank in order to consistently guide the lead weights over the center of each refuge. In the field setting, a PVC frame held a guiding section of pipe, to standardize and center the weight to a 1 m drop over the patch reef. The length of each line was calibrated to prevent the weight from hitting the substrate in order to reduce auditory or vibration cues. In the field setting, the weight was dropped while underwater, so it lacked the auditory and visual cues from the weight breaking the surface of the water. However, all fish in the field responded in some way to the weight drop. Observers triggered the release from either behind a blind (small aquaria setting) or from at least 1.5 m away (large aquaria and field settings).

Distances were estimated visually. A pilot study revealed visual estimates were within 10% of actual distances measured with a ruler. Two observers collected the data used in this study; both were trained by a more experienced observer. During training, inter-observer variation was less than 10% for all behavioral measures. Observations of behavior were aided by the use of a magnifying glass (2×). An observation time of 3 min was used to assess behavior since McCormick and Meekan (2010) found that this period produced low coefficients of variation (0–0.15) in behavioral observations [23]. Their study demonstrated consistency in behaviors among three consecutive 3 min observations, and White et al. (*in prep.*) demonstrated behavioral consistency of individuals (using the variables measured in our study) in a field situation over 2–3 days.

### Data Analysis

Height from substrate was recorded as the percentage of time an individual fish spent on the bottom (B), middle (M), or top (T) portions of the coral or PVC refuge. These values were transformed to a single, continuous mean variable using the formula: (B × 0/100)+(M × 5/100)+(T × 10/100). Similarly, distance ventured was recorded as the percentage of time spent 0 (A), 2 (B), 5 (C), or 10 (D) cm away from refuge. Data was transformed to a mean distance ventured using the formula: (A × 0/100)+(B × 2/100)+(C × 5/100)+(D × 10/100).

To examine whether individual fish changed behaviors across situations, but did so consistently, individuals were ranked and plotted for each behavioral trait and situation. Person’s product-moment correlations were compared across situations for each trait and were also used to identify relationships between specific traits, as well as behavioral traits and water temperature for each situation.

Principal components analysis (PCA) was used to examine the inter-relationships of behaviors (bite rate, distance moved, distance ventured, height, boldness, latency, and aggression) and individual behavioral consistency across each situation. Parallel analysis was used to determine the number of factors to be extracted (using permutations of 1000 parallel generated datasets) [37]. With the correct number of factors determined by the parallel analysis, principal component loadings were calculated using a correlation matrix with Direct Oblimin rotation [37]. Hierarchical agglomerative cluster analysis [38] for the 7 behavioral variables was overlaid with the PCA in order to determine if fish behaved similarly within each situation. Euclidean distance and unweighted pair-group method using arithmetic averages (UPGMA) were used to calculate clusters. For the PCA and Pearson’s product-moment correlations, the seven traits were log_10_ (x+1) transformed to improve normality. Analyses used SPSS [39] software.

## Results

A pilot study revealed that fish in the field began to explore their environment and feed within 1 minute of release onto patch reefs. Fish released into aquaria needed 20 mins to a few hours before exhibiting similar behavior (large and small aquaria respectively). The quicker acclimation time suggested that fish were less stressed and naturally inclined to start exhibiting “normal” behaviors in the field. There was no effect of time of day or observer on observed behavioral measures. All behavioral traits were highly variable both within and among individuals among settings.

### Consistency among Situations

There was no clear pattern of rankings of individuals for any of the behaviors such as boldness, distance moved and bite rate across situations (Fig. 1). A fish ranked highly for these traits in the small tank was just as likely to be medium or low ranked in the large tank and field situations. There was a significant positive correlation in height scores between small tanks and the field (r = 0.48, p = 0.004).

### Situation-dependent Relationships among Behaviors

**Figure 2:**
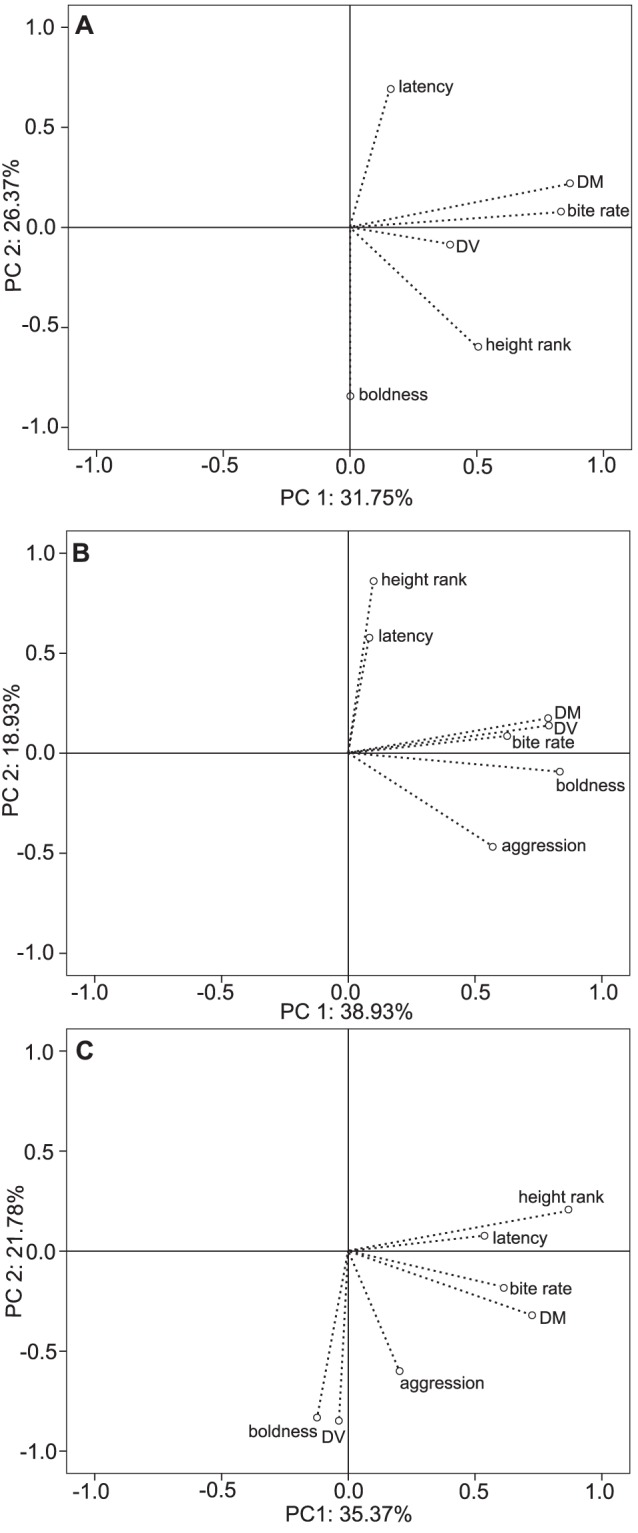
Principal component analysis of relationships between 7 behavioral traits. Traits include: bite rate, distance moved, distance ventured, height, boldness, latency and aggression in *P. amboinensis* in A) small tanks, B) large tanks and C) field situations.

**Figure 3:**
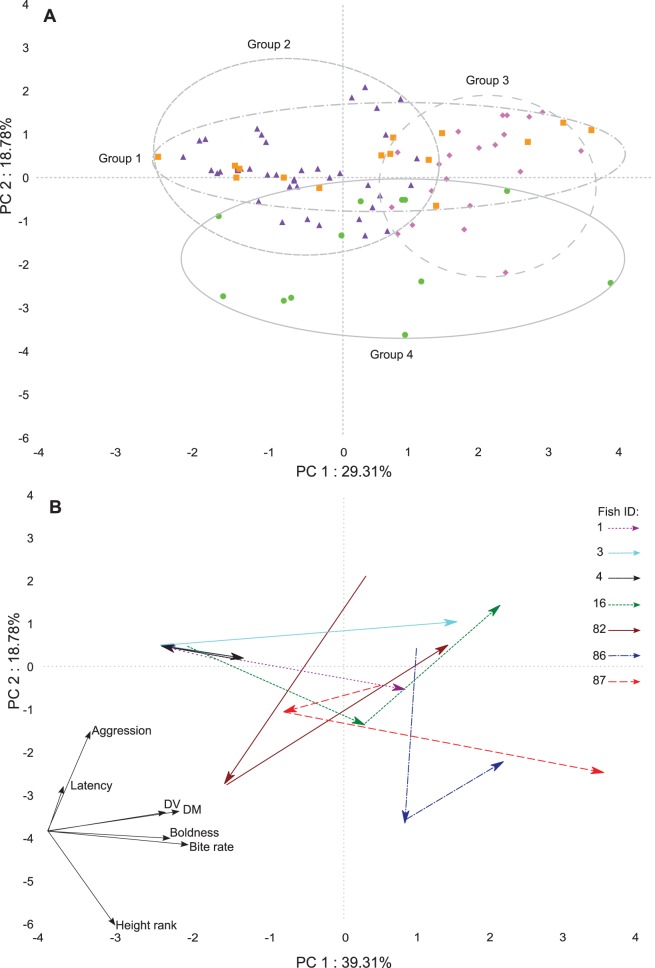
Principal component analysis of 7 behavioral traits for individual fish. A) PCA traits include: bite rate, distance moved, distance ventured, height, boldness, latency and aggression for individual fish (N = 33) across small tanks, large tanks, and field site (Total N = 99). Factor loadings of these traits represented by arrows in lower left corner. Ovals represent groupings created by clustering analysis. 67% of replicates in group 1 (square symbol) were from observations made in large tanks. 56% of replicates in group 2 (triangle symbol) were from small tanks. Group 3 (diamond symbol) is comprised of 76% of large tank observations. Group 4 (circle symbol) is composed of 42% large tank observations. Group 4 and the combination of groups 1, 2, and 3 represent the first split in the hierarchy. B) Identical principal components analysis with fish plots removed. Arrows represent factor loading patterns for seven randomly chosen fish from small tanks, large tanks, and field site.

Principal component analysis showed distance moved and bite rate had similar loadings across PC1 for each situation (Fig. 2; Table S1). Other relationships between behavioral traits differed among situations (Figs 2 & 3). In small tanks, boldness and latency had nearly opposite loadings over PC2 (Fig. 2a), which differed across situations. In large tanks, distance moved, distance ventured, and boldness scores had similar correlations with PC1. Height rank and latency also showed similar loadings over PC2 (Fig. 2b). In the field, boldness and distance ventured had similar loadings on both PC1 and PC2 (Fig. 2c). Height rank and latency had similar loadings, yet with different strengths of correlation with PC1 and PC2 for observations within large tanks and in the field (Figs. 2b & c).

Hierarchical cluster analysis created overlapping groups when superimposed on the principal components analysis. Groups did not separate clearly by situation (Fig. 3a). We did not find any consistency in behavioral traits when comparing a single situation at a time (Fig. 2) or across all situations at once (Fig. 3b). Correlation analysis showed a pattern of an increasing number of significant correlations among measured behavioral traits from small tanks, field site, to the large tanks (Table S2). In the small tank and field situations, there were strong significant correlations between bite rate and distance moved. In large tanks, distance moved was correlated with distance ventured. Also, boldness was positively correlated with distance ventured. While in the field, bite rate was positively related to distance moved. Fish which ventured a greater distance also tended to have higher scores of boldness.

### Effects of Temperature

There were no consistent correlations across situations for the relationship between behavioral traits and temperature. There was a positive relationship between temperature and bite rate, and height rank in the small tanks. Aggression was negatively related to water temperature in the large tanks while distance ventured and maximum distance ventured were negatively correlated in the field. However, none of the relationships were significant after Holm’s sequential Bonferonni adjustment [38]. Overall, temperatures averaged 29°C, ranging between 24–33.6°C, however this varied slightly among situations (Table 1).

**Table 1:** Average temperature ranges during behavioral assessments within each situation (small tank, large tank, field situation).

	Temperature (°C)			
Situation	Mean	Min.	Max.	Range
Small tank	29.1	24.1	33.0	8.9
Large tank	29.4	24.6	33.6	9.0
Field	29.0	28.6	30.5	1.9

Values of P do not control for multiple testing of the same data (*P<0.05; **P<0.01; ***P<0.001). Only values printed in bold are significant after Holm’s sequential Bonferonni adjustment of experimental error rates (Quinn and Keough 2002).

## Discussion

A behavioral syndrome across individuals can appear as consistent trends in the direction of loadings in a principal component analysis and can also be demonstrated among individuals by multiple significant correlations among the same behavioral traits across situations [1]. *P. amboinensis* did not show any evidence of a behavioral syndrome (i.e. a suite of correlated behaviors across situations) based on these analyses, although the lack of behavioral stability is not necessarily surprising.

An important result of our study was the lack of consistency in the rank order of all behavioral traits for individual *P. amboinensis* across the three situations (small tanks, large tanks, field site). The definition of behavioral syndromes accounts for this type of flexibility across situations [8] yet the premise of behavioral syndromes suggests some limitation of flexibility of behavioral responses [40]. Our results suggest that at this life history stageit is advantageous to remain highly flexible [1], [23] in behavior, rather than to develop syndromes. Young fish at settlement undergo high rates of mortality (averaging ∼ 60% within 48 hours, [26]) due to their small size and relatively poor competitive abilities [41] and they must be prepared to adapt rapidly to novel conditions. For these reasons, the ability to alter behavior to suit the new challenges they face may be key to survival.

White et al. (*in prep.*) recorded consistent individual rankings in scores of boldness, distance moved and occupancy height of reefs for newly-settled *P. amboinensis* over a three day period in the field. This finding suggests that behavioral patterns can be highly variable across different situations, yet at the same time show consistency within a single situation. Similarly, Coleman and Wilson (1998) found consistent individual boldness scores in two different contexts, but no correlation across contexts in juvenile pumpkinseed sunfish (*Lepomis gibbosus*) [42]. This implies that behavioral studies may have limited predictive ability when expanded to other situations; a finding that may be particularly relevant to laboratory-based work. Artificial environments can introduce variation in behavior due to confounding factors such as handling stress or experiences gained from life in captivity [14]. For example, farmed fish that live in an environment of high competition and no predation pressure are often bolder, more aggressive and take more risks than their wild counterparts [43], [44]. This idea was also supported by Wilson et al. (1993) who found individual boldness to be stable in nature but absent in the laboratory for juvenile pumpkinseed sunfish (*L. gibbosus*). They argued local environmental conditions maintain differences between individual behavioral phenotypes [45]. Our pilot study revealed that fish in the field have a reduced acclimation time compared to those held in aquaria, suggesting they are less stressed and naturally inclined to start exhibiting “normal” behaviors more quickly in the field. Studies in the field also have the added benefit of incorporating realistic environmental and ecological factors that may influence behavior (e.g. quantifying the ecological trade-offs of individual variation in behavior).

There were no significant correlations between behavioral traits and temperature for each of the three situations after correcting for multiple comparisons. Thus, despite large fluctuations in daily water temperature (up to 9°C and 2°C in the lab and field respectively), temperature did not meaningfully affect behavioral traits in any consistent manner. In a laboratory study using 6 L tanks filled halfway, Biro *et al.* (2010) found average values for activity, boldness and aggressiveness to increase by a factor of 2.5 to 6 in two species of damselfish in response to daily water temperature fluctuations of 3°C or less [16]. McCormick and Meekan (2010) also found a significant positive relationship between activity and temperature in the field for *P. amboinensis* [23]. Metabolic rate has been shown to increase exponentially with temperature in other ectotherms [46], and individual differences in metabolism are thought to be contribute to individual differences in behavioral traits for these animals [6], [47]. In our study, bite rate showed a positive correlation (however this was not significant after Bonferonni adjustment) with temperature within small tanks (over twice as large as the aquaria used by Biro *et al.* 2010), which would seem to agree with Biro *et al*.’s (2010) findings. Perhaps if behavioral observations in the present study were conducted following the protocol of Biro *et al.* (2010) (only one situation, in very small aquaria), our results might concur. In any event, it is clear that correlations developed from laboratory studies require validation in field environments in order to confirm that they have real ecological meaning.

The most consistent relationships among behaviors across situations were the close positive relationship between bite rate and distance moved across both the principal components (PC1 & PC2). Similarly, the correlation analysis showed positive correlations between bite rate and distance moved across small tank and field situations. These relationships are intuitive because individuals move more often and at greater distances when in pursuit of planktonic food. These relationships probably reflect the active foraging style of damselfish. All other relationships between behavioral traits were inconsistent across all situations. This result agrees with previous work on sticklebacks, which found no consistency in boldness, aggression, and activity across different situations between two populations [17]. This suggests behavioral syndromes do not always fit within the theory of the “constraint hypothesis” [48] which states behavioral syndromes are derived from a shared link between behaviors and assumes that the decoupling mechanisms underlying correlated behavioral traits do not evolve readily, because they would require changes in hormonal machinery [17]. The different relationships of behavioral traits among situations suggests either that there are biotic or abiotic factors that influence certain traits in different situations [17] or that the traits themselves can represent different things in different situations (e.g. the same measurement of boldness, or propensity to take risks, across different situations may in fact represent different traits due to how the individual perceives risk across the different situations).

It has been assumed that consistent individual differences in behavioral traits can occur due to differences in underlying physiological, behavioral, or morphological characteristics (i.e. state variables; [2], [49]) and that these variables establish the efficiency of certain types of behavior [5]. For example, if predation risk is a function of body size, and since body size is stable over short time scales (daily), animals of different body sizes should differ consistently with respect to their tendency to take risks while foraging. Therefore, theory predicts behavioral patterns related to body size should also be stable over the same time frame [2], [50]. The same logic applies to any other behavioral pattern linked to underlying state variables that are stable over time but vary among individuals [5]. While state variables are likely important in establishing stable behaviors, individual fish can rapidly respond to environmental factors that influence the behavioral patterns displayed. Thus, environmental factors are likely to be just as important in developing or maintaining stable behavioral syndromes. Behavioral flexibility is likely necessary for fish to quickly adjust to a completely different environment once they leave their pelagic larval phase. Bell and Sih (2007) found only populations of sticklebacks raised under strong predation pressure developed a correlative relationship between aggressiveness and boldness [51], demonstrating selective mortality and/or experience can help shape and establish behavioral patterns. Coral reef fishes may need some exposure to predators and environmental experience before developing a consistent behavioral syndrome.

In this study, naïve newly settled juveniles of *P. amboinensis* were found to lack consistency in: i) the rank order of behaviors across situations; ii) the relationship between behaviors and water temperature; iii) correlations in behaviors across situations. Given our results, it is interesting to note the lack of multi-situation or setting comparisons in previous studies that are often cited as evidence for the existence of behavioral syndromes. There have been a small number of mark-recapture [52] or short term (days) repeated studies (White et al., *in prep.*) that have properly met the definition of behavioral syndromes; yet the vast majority of fish studies only measure one behavioral trait, or show correlation under a single situation/context. Evidence for behavioral syndromes and personality in fish is weakened by the lack of multi-situation or multi-context comparisons. For example, boldness is defined as risk-taking and aggression as agonistic interactions between individuals and correlations between these two traits are often cited as a demonstration of behavioral syndromes. However, the operational definition and methods used to assess boldness are quite varied [53]. In some instances, aggressive interactions could also be considered risky. In some previous studies, this relationship could be an artifact of overlapping behavioral traits, where boldness and aggression are characteristics of a single behavioral trait rather than two distinct traits, or alternatively inadequate measures, if methods used to assess boldness actually assess both boldness and aggression. These issues cannot be resolved from observations made in a single situation. Comparisons across multiple situations (ideally including a natural setting) are necessary to establish within-individual or between-individual behavioral syndromes that by Sih et al.’s (2004) definition suggest some degree of situation or context independence.

## Supporting Information

Table S1 **Factor components from PCA of Behaviors in each situation.**
(DOCX)

Table S2 **Correlations between 8 behavioral traits of** ***P. amboinensis*** **in A) small tanks, B) large tanks, and C) field site.** Only significant values are presented (p<0.05). DM = distance moved, DV = distance ventured. † Values of P do not control for multiple testing of the same data (*P<0.05; **P<0.01; ***P<0.001). Only values printed in bold are significant after Holm’s sequential Bonferonni adjustment of experimental error rates (Quinn and Keough 2002).
(DOCX)
