# Supplementary material for: Syndromes or Flexibility: Behavior during a Life History Transition of a Coral Reef Fish
Source: PLoS One. 2013 Dec 27;8(12):e84262. doi: 10.1371/journal.pone.0084262 (PMC3874005; doi:10.1371/journal.pone.0084262)
Supplement: Table S1 — Factor components from PCA of Behaviors in each situation. (DOCX) [file pone.0084262.s001.docx]

|  | **Component** | |
| --- | --- | --- |
|  | **1** | **2** |
| **Small tank** |  |  |
| Bite rate | 0.832 | 0.079 |
| Distance moved | 0.868 | 0.220 |
| Distance ventured | 0.395 | -0.086 |
| Height rank | 0.506 | -0.596 |
| Boldness | 0.002 | -0.843 |
| Latency | 0.161 | 0.692 |
|  |  |  |
| **Large tank** |  |  |
| Bite rate | 0.627 | 0.086 |
| Distance moved | 0.788 | 0.175 |
| Distance ventured | 0.791 | 0.139 |
| Height rank | 0.100 | 0.861 |
| Boldness | 0.834 | -0.092 |
| Latency | 0.083 | 0.578 |
| Aggression | 0.570 | -0.468 |
|  |  |  |
| **Field** |  |  |
| Bite rate | 0.614 | -0.184 |
| Distance moved | 0.725 | -0.321 |
| Distance ventured | -0.037 | -0.848 |
| Height rank | 0.869 | 0.208 |
| Boldness | -0.123 | -0.832 |
| Latency | 0.537 | 0.077 |
| Aggression | 0.203 | -0.600 |

**Table S1.**
