# Supplementary material for: Syndromes or Flexibility: Behavior during a Life History Transition of a Coral Reef Fish
Source: PLoS One. 2013 Dec 27;8(12):e84262. doi: 10.1371/journal.pone.0084262 (PMC3874005; doi:10.1371/journal.pone.0084262)
Supplement: Table S2 — Correlations between 8 behavioral traits of P. amboinensis in A) small tanks, B) large tanks, and C) field site. Only significant values are presented (p<0.05). DM = distance moved, DV = distance ventured. † Values of P do not control for multiple testing of the same data (*P<0.05; **P<0.01; ***P<0.001). Only values printed in bold are significant after Holm’s sequential Bonferonni adjustment of experimental error rates (Quinn and Keough 2002). (DOCX) [file pone.0084262.s002.docx]

**Table S2.**

| A) †Small tank | Bite rate | DM | DV | Height rank | Boldness | Latency | Aggression |
| --- | --- | --- | --- | --- | --- | --- | --- |
| Bite rate |  | **0.54**** |  |  |  |  |  |
| DM |  |  |  |  |  |  |  |
| DV |  |  |  |  |  |  |  |
| Height rank |  |  |  |  | 0.44* |  |  |
| Boldness |  |  |  |  |  |  |  |
| Latency |  |  |  |  |  |  |  |
| Aggression |  |  |  |  |  |  |  |

| B) †Large tank | Bite rate | DM | DV | Height rank | Boldness | Latency | Aggression |
| --- | --- | --- | --- | --- | --- | --- | --- |
| Bite rate |  | 0.49** | 0.38* |  | 0.41* |  |  |
| DM |  |  | **0.61***** |  | 0.44* |  |  |
| DV |  |  |  |  | **0.64***** |  |  |
| Height rank |  |  |  |  |  |  |  |
| Boldness |  |  |  |  |  |  | 0.45** |
| Latency |  |  |  |  |  |  |  |
| Aggression |  |  |  |  |  |  |  |

| C) †Field site | Bite rate | DM | DV | Height rank | Boldness | Latency | Aggression |
| --- | --- | --- | --- | --- | --- | --- | --- |
| Bite rate |  | **0.52**** |  | 0.44** |  |  |  |
| DM |  |  |  | 0.42* |  |  | 0.50** |
| DV |  |  |  |  | **0.56***** |  | 0.39* |
| Height rank |  |  |  |  |  | 0.35* |  |
| Boldness |  |  |  |  |  |  |  |
| Latency |  |  |  |  |  |  |  |
| Aggression |  |  |  |  |  |  |  |
